# Brain Oxygen Extraction and Metabolism in Pediatric Patients With Sickle Cell Disease: Comparison of Four Calibration Models

**DOI:** 10.3389/fphys.2022.814979

**Published:** 2022-02-11

**Authors:** Zixuan Lin, Tiffany McIntyre, Dengrong Jiang, Alicia Cannon, Peiying Liu, Aylin Tekes, James F. Casella, Keith Slifer, Hanzhang Lu, Eboni Lance

**Affiliations:** ^1^The Russell H. Morgan Department of Radiology and Radiological Science, The Johns Hopkins University School of Medicine, Baltimore, MD, United States; ^2^Department of Neurology and Developmental Medicine, Kennedy Krieger Institute, Baltimore, MD, United States; ^3^Department of Neuropsychology, Kennedy Krieger Institute, Baltimore, MD, United States; ^4^Division of Pediatric Hematology, Department of Pediatrics, The Johns Hopkins University School of Medicine, Baltimore, MD, United States; ^5^Department of Behavioral Psychology, Kennedy Krieger Institute, Baltimore, MD, United States; ^6^Department of Psychiatry and Behavioral Sciences, The Johns Hopkins University School of Medicine, Baltimore, MD, United States; ^7^F. M. Kirby Research Center for Functional Brain Imaging, Kennedy Krieger Institute, Baltimore, MD, United States; ^8^Department of Neurology, The Johns Hopkins University School of Medicine, Baltimore, MD, United States

**Keywords:** sickle cell disease, OEF, CMRO_2_, CBF, TRUST MRI

## Abstract

Sickle cell disease (SCD) is an inherited hemoglobinopathy with an increased risk of neurological complications. Due to anemia and other factors related to the underlying hemoglobinopathy, cerebral blood flow (CBF) increases as compensation; however, the nature of alterations in oxygen extraction fraction (OEF) and cerebral metabolic rate of oxygen (CMRO_2_) in SCD remains controversial, largely attributed to the different calibration models. In addition, limited studies have been done to investigate oxygen metabolism in pediatric patients. Thus, this study used a non-invasive T_2_-based MR oximetry, T_2_-Relaxation-Under-Spin-Tagging (TRUST) MRI, to measure oxygen homeostasis in pediatric patients with SCD using four different calibration models and examined its relationship to hematological measures. It was found that, compared with controls, SCD patients showed an increased CBF, unchanged total oxygen delivery and increased venous blood T_2_. The results of OEF and CMRO_2_ were dependent on the calibration models used. When using sickle-specific, hemoglobin S (HbS) level-dependent calibration, there was a decreased OEF and CMRO_2_, while the bovine model showed an opposite result. OEF and CMRO_2_ were also associated with hemoglobin and HbS level; the direction of the relationship was again dependent on the model. Future studies with *in vivo* calibration are needed to provide more accurate information on the T_2_-Y_*v*_ relationship.

## Introduction

Sickle cell disease (SCD) is an inherited blood disorder, in which a beta globin gene mutation causes the formation of a mutated protein, hemoglobin S (HbS), leading to the deformation of red blood cell (RBC) into a sickled shape. This also leads to hemolysis and decreased hemoglobin, thus decreased arterial oxygen content, resulting in an elevated cerebral blood flow (CBF) in order to compensate for the oxygen demand of the brain (Herold et al., 1986; Hurlet-Jensen et al., 1994a; Li et al., 2020). SCD has been associated with a number of brain physiological disorders, including increased cerebral vasculopathy, reduced cerebrovascular reactivity, and increased risk of overt stroke or silent cerebral infarction (SCI) (Pauling et al., 1949; Hurlet-Jensen et al., 1994b; Miller et al., 2001; Prengler et al., 2002; Switzer et al., 2006; Leung et al., 2016; Juttukonda et al., 2017; Bush et al., 2018a; Jordan and DeBaun, 2018; Václavů et al., 2019; Jacob et al., 2020; Kirkham and Lagunju, 2021; Stotesbury et al., 2021). An increased risk of neurodevelopmental disorders in pediatric SCD patients was also reported (Berkelhammer et al., 2007; Lance et al., 2015, 2021).

One potential physiological biomarker in SCD is the oxygen extraction fraction (OEF). Due to the lack of oxygen carrying capacity of the sickled blood cell, the OEF may increase as a compensation; however, when the CBF and flow velocity increase, the OEF may also decrease due to insufficient diffusion time (Juttukonda et al., 2020) or excessive blood flow (Bush et al., 2018b) in arteriovenous shunting. Meanwhile, cerebral metabolic rate of oxygen (CMRO_2_), which represents the oxidative metabolism of the brain, may also be altered. Additionally, increased OEF has been associated with increased risk of stroke in certain regions of the brain (Fields et al., 2018). Chronic blood transfusion, a disease modifying treatment for SCD, is associated with lower CBF and OEF (Guilliams et al., 2018).

The gold standard to measure the OEF and CMRO_2_ is ^15^O-PET, which is highly invasive and requires onsite production of ^15^O isotope. Herold et al. (1986) using ^15^O-PET reported no significant differences in OEF (42 ± 4% for control and 44 ± 7% for SCD) or CMRO_2_ between SCD and control groups, while Heyman et al. (1952) using a nitrous oxide method showed moderately reduced arteriovenous oxygen difference and CMRO_2_ in SCD. Recently, several MRI-based techniques have been developed to measure OEF non-invasively. One is T_2_-based oximetry, based on the principle that oxygen content in the blood can alter the blood T_2_ due to its magnetic property (Wright et al., 1991; Silvennoinen et al., 2003; Zhao et al., 2007; Lu and Ge, 2008; Lu et al., 2012). Several studies have been conducted in SCD patients to investigate the role of OEF and CMRO_2_ using the T_2_-based method (Jordan et al., 2016; Bush et al., 2018b,^2021^; Morris et al., 2018; Vaclavu et al., 2018; Juttukonda et al., 2019, 2020; Li et al., 2020; Václavů et al., 2020; Prussien et al., 2021; Vu et al., 2021); however, the results of these studies are variable, largely due to the calibration models used to convert venous blood T_2_ to venous oxygenation (Y_*v*_). Using a bovine blood model, Jordan et al. (2016) reported increased OEF and CMRO_2_ in SCD; however, it has been speculated that the bovine model may not be suitable for sickled blood, due to a much lower hematocrit range and different size, shape, and permeability of the sickled blood cell (Clark and Rossi, 1990; Gibson and Ellory, 2002). Thus, Bush et al. (2018b) reported a HbS calibration model which showed a decreased OEF and CMRO_2_ in SCD. However, the Bush model has no hematocrit dependence and omitted the linear term in the equation, yielding an OEF value approximately half of the results from the PET study. In addition, Li et al. (2020) presented an individual calibration model and reported no change in OEF and CMRO_2_ in SCD. In order to establish a unified model for sickled blood, Li’s group and Bush’s group combined their data and proposed a Li-Bush sickle-specific calibration model (Bush et al., 2021).

No studies have been done to compare all these four models in one cohort. In addition, the majority of the T_2_-based studies mentioned above were conducted in adult SCD patients. The oxygen metabolism in pediatric patients may be different than in adults. Thus, in this study, we aimed to investigate the oxygen homeostasis in a group of pediatric SCD participants, using T_2_-Relaxation-Under-Spin-Tagging (TRUST) MRI (Lu and Ge, 2008; Lu et al., 2012; Xu et al., 2012; Jiang et al., 2018). Results from all four available T_2_-Y_*v*_ calibration models were compared. Difference in oxygen homeostasis between SCD participants and age-matched controls were examined. The relationships between oxygen homeostasis and different SCD phenotypes, hematological measures, silent cerebral infarction and neuropsychological performance were also examined.

## Materials and Methods

### Participants

The Institutional Review Board (IRB) of Johns Hopkins University approved the study and the guardians of the participants signed IRB-approved consent forms before enrolling in the study. A total of 28 pediatric participants were recruited (10.0 ± 1.3 years, 12M/16F), including 21 SCD participants and seven controls. The inclusion criteria for SCD participants was 8–12 years of age with diagnosis of SCD validated by lab testing or confirmed by hematologist. Participants with any prior history of stroke, silent cerebral infarction or seizures, participants who need an interpreter, and participants who were adopted or in foster care were excluded. Study recruitment of sickle cell disease patients was done through local pediatric hematology clinics. Siblings of SCD participants were recruited as control participants. Additional control participants were recruited through internet postings, flyers, and email. The study recruitment started from May 2018 and ended in June 2021. Among SCD participants, 11 had hemoglobin SS, one had hemoglobin Sβ^0^ thalassemia, three had hemoglobin Sβ^+^ thalassemia and six had hemoglobin SC. Fourteen of the SCD participants were on hydroxyurea treatment in the past but not at the time of study. Among the seven controls, four were siblings of SCD patients and had sickle cell trait (HbS fraction range from 31.9 to 39.2%, median 33.1%). 8 out of the 21 SCD participants had abnormal MRI findings, including six with SCI, one with few scattered white matter hyperintensities and one with hypothalamic/thalamic mass.

### Hematological Assessment

Blood samples were obtained for all participants on the day of the MRI. Concentration of hematocrit, hemoglobin and reticulocytes were determined for all participants. Fraction of HbS was measured for all SCD participants and a subset of three controls with sickle cell trait. One trait participant has HbAC trait, thus no HbS value.

### MRI Experiments

All participants were studied on a 3T Philips Ingenia System (Philips Healthcare, Best, Netherlands).

**FIGURE 1:**
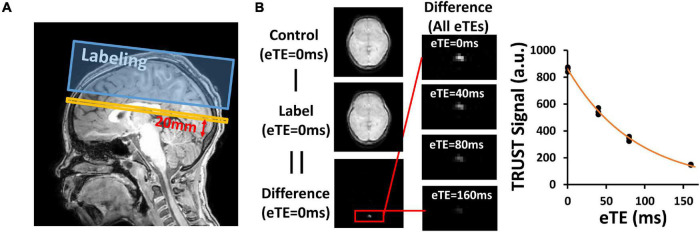
Representative results of T_2_-Relaxation-Under-Spin-Tagging (TRUST) MRI. **(A)** Position of image plane for TRUST MRI. Yellow bar indicates the imaging slice. Blue box indicates the labeling slab. **(B)** Representative images of TRUST MRI for quantification of venous blood T_2_. Paired subtraction between control and label images results in a different image, in which the superior sagittal sinus (red box) is prominent. Fitting of the signal as a function of eTE results in the estimation of venous blood T_2_.

T_2_-Relaxation-Under-Spin-Tagging MRI was performed for the measurement of OEF by isolating the venous blood signal with venous spin labeling, followed by a series of T_2_ preparation pulses (Lu and Ge, 2008; Xu et al., 2012; Figure 1A). The imaging parameters were as follows: TR = 3,000 ms, TE = 3.61 ms, inversion time (TI) = 1022 ms, flip angle = 90°, FOV = 220 mm × 220 mm × 5 mm, voxel size = 3.44 mm × 3.44 mm × 5 mm, four eTEs (0, 40, 80, and 160 ms) with a τCPMG of 10 ms, labeling thickness = 100 mm, and scan duration = 1.2 min.

Phase-contrast (PC) MRI was then performed to measure the global CBF (Peng et al., 2014, 2015). An angiogram was first conducted at the cervical region to visualize the four feeding arteries (left and right internal carotid arteries, left and right vertebral arteries), with the following parameters: TR = 26 ms, TE = 5.8 ms, flip angle = 20°, FOV = 200 mm × 200 mm × 80 mm, voxel size = 0.5 mm × 0.99 mm × 2 mm, number of slices = 40, and scan duration = 1 min 39 s. PC MRI was acquired for each of the arteries with the imaging plane perpendicular to the targeting artery. The imaging parameters were as follows: TR = 18.9 ms, TE = 9.3 ms, flip angle = 15°, FOV = 200 mm × 200 mm × 5 mm, voxel size = 0.5 mm × 0.5 mm × 5 mm, single slice, encoding velocity (V_*enc*_ = 40 cm/s), and scan duration = 15 s.

A T_1_-weighted magnetization-prepared-rapid-acquisition-of-gradient-echo (MPRAGE) scan was acquired for brain volume quantification with the following parameters: radial acquisition, TR = 7.2 ms, TE = 3.1 ms, shot interval = 2,150 ms, TI = 1,100 ms, flip angle = 10°, FOV = 224 mm × 224 mm × 160 mm, voxel size = 1 mm × 1 mm × 1 mm, number of slices = 160, sagittal orientation, and scan duration = 2 min 28 s.

### MRI Data Processing

To obtain OEF, subtraction between TRUST control and label images yields pure venous blood signals. Region of interest (ROI) containing the SSS were drawn manually on the difference images and the four voxels with maximum signal intensity were chosen. Venous blood T_2_ was quantified by fitting the signals at different effective echo times to a mono-exponential model (Figure 1B). Blood T_2_ was then converted to Y_*v*_ using four available calibration models:

(1) A bovine model (Lu et al., 2012).

$$\frac{1}{T_{2}}=A+B\cdot \left(1-Y\right)+C\cdot {\left(1-Y\right)}^{2}$$

Where *A* = −13.5 + 80.2 ⋅ *Hct*−75.9 ⋅ *Hct*^2^, *B* = 0.5 ⋅ *Hct* + 3.4 ⋅ *Hct*^2^, and *C* = 247.4 ⋅ *Hct* ⋅ (1−*Hct*).
(2) A sickle human blood model calibrated with HbS blood (Bush model) (Bush et al., 2018b).

$$\frac{1}{T_{2}}=A\cdot {\left(1-Y\right)}^{2}+B$$

Where *A=70* and *B* = 5.75.
(3) A sickle blood model calculated based on individual calibration (Li model) (Li et al., 2020) (detailed parameters was obtained from the authors through personal communication).

$$\frac{1}{T_{2}}=A+B\cdot \left(1-Y\right)+C\cdot {\left(1-Y\right)}^{2}$$

Where *A* = 21.7−108.5 ⋅ *Hct* + 189.5 ⋅ *Hct*^2^, *B* = 56.2 ⋅ *Hct*−73.1 ⋅ *Hct*^2^, and *C* = 242.5 ⋅ *Hct* ⋅ (1−*Hct*).
(4) A joint model calculated based on the datasets from the previous two studies (Li-Bush model) (Bush et al., 2021).

$$\frac{1}{T_{2}}=A\cdot {\left(1-Y\right)}^{2}+B$$

Where *A* = 196.8 ⋅ *Hct* + 16.7 and *B* = −6.6 ⋅ *Hct* + 8.6.

For the last three models, to account for subject-specific HbS level, the respective sickled blood model and normal human blood model (as stated in each of these studies) were first interpolated to obtain a Hb-specific calibration curve, i.e., a linear mixture of the HbS and normal human blood calibration weighted by individual HbS level, from which Y_*v*_ was estimated. OEF was calculated using the following equation:


(1) $$O\,E\,F=\left(Y_{a}-Y_{v}\right)/Y_{a}$$

Where Y_*a*_ is arterial oxygenation (assumed to be 98%).

For PC-MRI, ROIs were drawn manually for the left/right internal carotid arteries and left/right vertebral arteries. The total flux of the arteries were calculated by the sum of the velocity in the ROI (Peng et al., 2015). Aliasing correction was done when the maximum velocity exceeded the VENC. The T_1_-MPRAGE images were segmented using an automatic processing tool, MRICloud (Mori et al., 2016) (Johns Hopkins University, Baltimore, MD, United States)^1^ for brain volume quantification. Then the global CBF was quantified by normalizing the total flux with the total brain parenchymal volume.

The oxygen delivery rate DO_2_ (μmol/100 g/min) was calculated based on individual hematocrit level:


(2) $$D\,O_{2}=C\,B\,F\cdot C\,h\cdot Y_{a}$$

Where Ch is amount of oxygen that a unit of volume of blood can carry and is Hb dependent. In this study, it was assumed to be Ch = 20.4*hematocrit, based on previous literature (Guyton and Hall, 2005). CMRO_2_ was also calculated based on Fick Principle (Kety and Schmidt, 1948):


(3) $$C\,M\,R\,O_{2}=C\,B\,F\cdot \left(Y_{a}-Y_{v}\right)\cdot C\,h$$

### Statistical Analysis

Age and education were compared between SCD participants and control participants by Wilcoxon rank sum test. Sex was compared between groups using Chi-square test. Group difference in hematological measures, brain volume and cognitive performance were examined using linear regression analysis, with age, sex, and education as covariates when relevant.

Cerebral physiological parameters, including CBF, DO_2_, OEF, and CMRO_2_ were compared between the SCD and control groups using linear regression analysis, where the physiological parameter was the dependent variable and group index was the independent variable, with age and sex as covariates. Considering that some of the control participants also had sickle cell trait, we further split the control subjects into two sub-groups and the above-mentioned parameters were compared between controls with trait and controls without trait.

To determine if the choice of the model affects the relative group differences or only the absolute values, relative change in OEF and CMRO_2_ was calculated as the percentage change in SCD compared to the average value in the control group. Then a Kruskal–Wallis test was used to compare the values among different models.

The association between the cerebral physiological parameters and the hematological measures were evaluated in the entire cohort using linear regression with the physiological parameter as the dependent variable, hematological parameter (i.e., hemoglobin, reticulocytes, or HbS) as the independent variable, age and sex as the covariates. Similar analysis was done within the SCD group as well.

In all analyses, a two-tailed *p*-value of 0.05 or less was considered statistically significant.

## Results

### Demographics and Hematological Measures

The demographic information for all participants were summarized in Table 1. There were no significant differences in age (*p* = 0.68), sex (*p* = 0.27) and education (*p* = 0.15) between the SCD and control groups. After controlling for age and sex, there were no differences in the brain volumes between two groups, including whole brain (*p* = 0.95), frontal lobe (*p* = 0.16), parietal lobe (*p* = 0.13), temporal lobe (*p* = 0.36) and occipital lobe (*p* = 0.63).

**TABLE 1:** Demographics information of SCD patients and control participants (mean ± SD).

	SCD	Control	*p*-value
*N*	21	7	
Age, years	9.9 ± 1.2	10.1 ± 1.7	0.68
Females, *N* (%)	11 (52%)	5 (71%)	0.27
Education, years	3.5 ± 1.3	4.4 ± 2.4	0.15
Hematocrit (%)	26.67 ± 5.4	37.69 ± 1.9	<0.0001
Hemoglobin (g/dL)	9.3 ± 2.0	12.8 ± 0.8	0.00027
HbS (%)	65.9 ± 16.9	15.7 ± 19.8	0.00074
Reticulocyte (%)	6.36 ± 4.58	1.16 ± 0.42	0.024
CBF (mL/100 g/min)	118.9 ± 27.7	73.0 ± 10.0	0.00043
*N* on hydroxyurea in the past	15		
*N* receive blood transfusion	0		
*N* for different SCD phenotypes	11 HbSS		
	1 HbSβ^0^		
	3 HbSβ^+^		
	6 Hb SC		

After controlling for age and sex in regression analysis, SCD patients had lower hematocrit (SCD: 26.7 ± 5.4%, control: 37.7 ± 1.9%, *p* < 0.0001), lower hemoglobin (SCD: 9.3 ± 2.0 gm/dL, control: 12.8 ± 0.8 gm/dL, *p* = 0.00027) and higher HbS fraction (SCD: 65.9 ± 16.9%, control: 15.7 ± 19.8%, *p* = 0.00074) than the control group.

### Cerebral Physiological Difference Between Sickle Cell Disease and Controls

**FIGURE 2:**
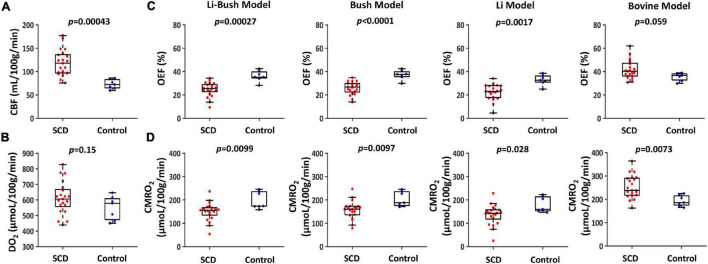
Cerebral physiological difference between SCD and controls. **(A)** Boxplot of CBF between SCD and control participants. **(B)** Boxplot of DO_2_ between SCD and control participants. **(C)** Boxplots of OEF calculated using four different calibration models between SCD and control participants. **(D)** Boxplots of CMRO_2_ calculated using four different calibration models between SCD and control participants.

Sickle cell disease participants had a significantly higher venous T_2_ compared with control participants (SCD: 91.2 ± 13.4 ms, control: 72.0 ± 9.6 ms, *p* = 0.0033, Table 2). Figures 2A,B showed the boxplots of CBF and DO_2_, respectively, in the SCD and control groups. Compared with control participants, SCD participants had a significantly higher CBF (SCD: 118.7 ± 27.7 mL/100 g/min, control: 73.0 ± 10.0 mL/100 g/min, *p* = 0.00043), but the total oxygen delivery DO_2_ was not significantly different between two groups (SCD: 612.3 ± 103.3 μmol/100 g/min, control: 549.5 ± 76.6 μmol/100 g/min, *p* = 0.15).

**TABLE 2:** Comparison of cerebral physiological parameters among different diagnostic groups.

	SCD	All controls	Controls: Trait	Controls: Non-trait	*p*-value between SCD and all controls	*p*-value between trait and non-trait
*N*	21	7	4	3		
CBF (mL/100 g/min)	118.7 ± 27.7	73.0 ± 10.0	69.1 ± 10.9	78.4 ± 7.0	0.00043	0.34
DO_2_ (mL/100 g/min)	612.3 ± 103.3	549.5 ± 76.6	508.5 ± 74.8	604.3 ± 36.8	0.15	0.22
T_2_ (ms)	91.2 ± 13.4	72.0 ± 9.6	74.1 ± 10.6	69.3 ± 9.4	0.0033	0.56
OEF-Li-Bush (%)	24.8 ± 6.1	36.2 ± 4.6	33.8 ± 4.0	39.4 ± 3.8	0.00027	0.22
OEF-Bush (%)	25.9 ± 5.4	37.2 ± 4.0	35.6 ± 3.8	39.4 ± 3.7	<0.0001	0.35
OEF-Li (%)	22.5 ± 6.7	32.8 ± 4.5	30.7 ± 4.2	35.7 ± 3.6	0.0017	0.28
OEF-Bovine (%)	41.8 ± 7.9	35.5 ± 3.4	35.1 ± 3.7	35.9 ± 3.7	0.059	0.77
CMRO_2_-Li-Bush (μmol/100 g/min)	150.0 ± 38.7	198.5 ± 36.7	169.6 ± 7.5	236.9 ± 8.9	0.0099	<0.0001
CMRO_2_-Bush (μmol/100 g/min)	157.3 ± 37.8	203.9 ± 31.9	179.1 ± 8.2	236.9 ± 8.9	0.0097	0.00070
CMRO_2_-Li (μmol/100 g/min)	136.8 ± 42.9	179.9 ± 33.1	153.9 ± 5.5	214.5 ± 9.4	0.028	0.00034
CMRO_2_-Bovine (μmol/100 g/min)	253.2 ± 49.1	193.6 ± 22.9	176.7 ± 9.8	216.2 ± 36.8	0.0073	0.0037

Figures 2C,D and Table 2 show OEF and CMRO_2_ when calibrated using different models. When using the Li-Bush model, SCD patients showed a significantly decreased OEF (*p* = 0.00027) and CMRO_2_ (*p* = 0.0099) compared with control participants. When using the Bush model, there was also a decrease in OEF (*p* < 0.0001) and CMRO_2_ (*p* = 0.0097) in SCD participants. Similar differences were found when using the Li-model (OEF: *p* = 0.0017; CMRO_2_: *p* = 0.028). But there were no significant differences in the relative change of OEF or CMRO_2_ in SCD compared to control group among these three models. In contrast, when using the bovine model, OEF showed an opposite result, i.e., a trend of increase in SCD participants compared with controls (*p* = 0.059), and CMRO_2_ were increased as well (*p* = 0.0073).

**FIGURE 3:**
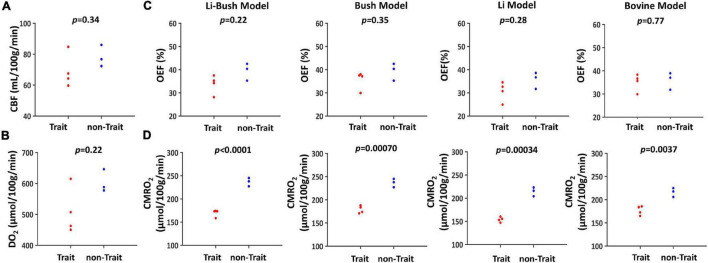
Cerebral physiological difference between trait and non-trait participants. **(A)** Swarmplot of CBF between trait and non-trait participants. **(B)** Swarmplot of DO_2_ between trait and non-trait participants. **(C)** Swarmplots of OEF calculated using four different calibration models between trait and non-trait participants. **(D)** Swarmplots of CMRO_2_ calculated using four different calibration models between trait and non-trait participants.

When dividing the controls into sickle cell S/C-trait and non-sickle cell S/C-trait participants, all four models showed that the trait participants had a lower mean CMRO_2_ compared with non-trait participants (Figure 3 and Table 2).

### Relationships Between Cerebral Physiology and Hematological Measures

**FIGURE 4:**
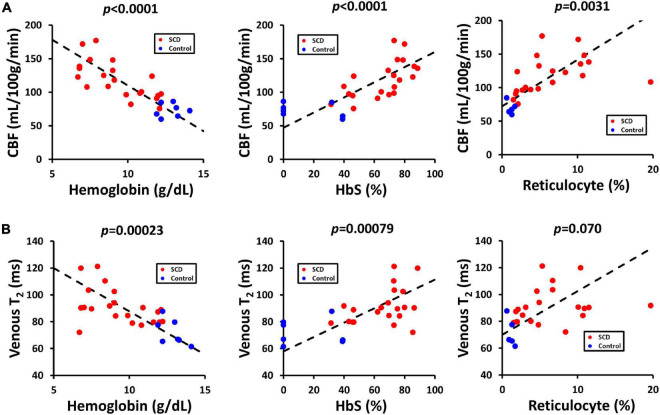
Relationships between CBF, venous T_2_, and hematological measures. **(A)** Scatterplots between CBF and hemoglobin, HbS and reticulocyte level. **(B)** Scatterplots between venous T_2_ and hemoglobin, HbS and reticulocyte level.

**FIGURE 5:**
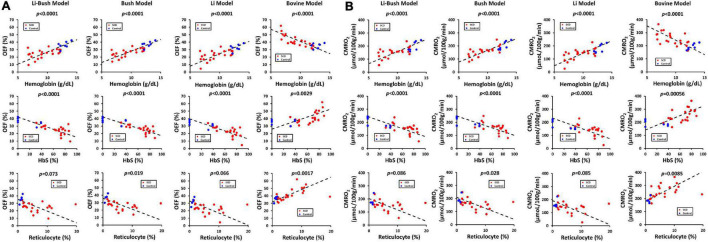
Relationship between OEF, CMRO_2_, and hematological measures. **(A)** Scatterplots between OEF from different models and hemoglobin, HbS and reticulocyte level. **(B)** Scatterplots between CMRO_2_ from different models and hemoglobin, HbS and reticulocyte level.

Table 3 showed the linear regression results between cerebral physiology and hematological measures. Linear regression analysis showed that the participants with lower hemoglobin had a significant higher CBF (β = −10.83, 95% CI [−13.95, −7.71], *p* < 0.0001) and a higher venous blood T_2_ (β = −4.25, 95% CI [−6.28, −2.23], *p* = 0.00023) (Figure 4). Results from the Bush model, Li model and Li-Bush model all indicated that lower hemoglobin level was associated with lower OEF and lower CMRO_2_ (Figure 5). However, the bovine model showed an opposite result, i.e., lower hemoglobin level was associated with higher OEF and higher CMRO_2_. There was no significant relationship between DO_2_ and hemoglobin level.

**TABLE 3:** Linear regression model results between cerebral physiology and hematological measures (entire cohort).

	Hemoglobin			HbS			Reticulocyte		
	Beta	*p*-value*	C.I.	Beta	*p*-value*	C.I.	Beta	*p*-value*	C.I.
CBF (mL/100 g/min)	–10.83	< 0.0001	[−13.95, −7.71]	1.06	< 0.0001	[0.68, 1.44]	3.68	0.0031	[1.39, 5.98]
DO_2_ (mL/100 g/min)	–1.50	0.8518	[−17.87, 14.87]	1.42	0.2671	[−0.40, 3.24]	–0.063	0.99	[−8.91, 8.79]
T_2_ (ms)	–4.25	0.00023	[−6.28, −2.23]	0.33	0.00079	[0.079, 0.58]	1.18	0.070	[−0.11, 2.47]
OEF-Li-Bush (%)	0.023	< 0.0001	[0.013, 0.033]	–0.0021	< 0.0001	[−0.0031, −0.0012]	–0.0057	0.073	[−0.012, 0.00057]
OEF-Bush (%)	0.025	< 0.0001	[0.018, 0.032]	–0.0022	< 0.0001	[−0.0030, −0.0014]	–0.0068	0.019	[−0.012, −0.0012]
OEF-Li (%)	0.024	< 0.0001	[0.014, 0.034]	–0.0023	< 0.0001	[−0.0033, −0.0013]	–0.0061	0.066	[−0.013, 0.00044]
OEF-Bovine (%)	–0.025	< 0.0001	[−0.034, −0.020]	0.0021	0.0029	[0.00078, 0.0033]	0.0099	0.0017	[0.0042, 0.016]
CMRO_2_-Li-Bush (μmol/100 g/min)	13.37	< 0.0001	[7.96, 18.78]	–0.83	< 0.0001	[−1.45, −0.21]	–3.03	0.086	[−6.52, 0.46]
CMRO_2_-Bush (μmol/100 g/min)	13.96	< 0.0001	[9.43, 18.49]	–0.85	< 0.0001	[−1.45, −0.26]	–3.66	0.028	[−6.88, −0.43]
CMRO_2_-Li (μmol/100 g/min)	13.74	< 0.0001	[8.19, 19.28]	–0.95	< 0.0001	[−1.62, −0.28]	–3.23	0.085	[−6.95, 0.49]
CMRO_2_-Bovine (μmol/100 g/min)	–14.61	< 0.0001	[−21.24, −7.97]	1.67	0.00056	[0.97, 2.38]	5.60	0.0085	[1.58, 9.63]

*Adjusted for age and sex.

It was also found that participants with a higher HbS fraction had a higher CBF (β = 1.06, 95% CI [0.68, 1.44], *p* < 0.0001) and higher venous T_2_ (β = 0.33, 95% CI [0.079, 0.58], *p* = 0.00079). When using the Bush model, Li-model or the Li-Bush model, participants with a higher HbS fraction showed a significant lower OEF and lower CMRO_2_. However, results from the bovine model showed an opposite result.

In addition, higher reticulocytes level was associated with higher CBF (β = 3.68, 95% CI [1.39, 5.98], *p* = 0.0031) and a trend of higher venous blood T_2_ (β = 1.18, 95% CI [−0.11, 2.47], *p* = 0.070). Results from the Bush model, Li model, and Li-Bush model all showed that participants with a higher reticulocytes count had a lower OEF and higher CMRO_2_, while the bovine model showed an opposite result. There was also no significant association between DO_2_ and reticulocytes level.

Similar analysis was done within the SCD patients only, and the results are largely consistent with the above findings in that entire cohort (Table 4).

**TABLE 4:** Linear regression model results between cerebral physiology and hematological measures (within SCD participants).

	Hemoglobin			HbS			Reticulocyte		
	Beta	*p*-value*	C.I.	Beta	*p*-value*	C.I.	Beta	*p*-value*	C.I.
CBF (mL/100 g/min)	–9.97	0.00015	[−14.32, −5.63]	1.02	0.0017	[0.44, 1.59]	2.55	0.051	[−0.016, 5.12]
DO_2_ (mL/100 g/min)	8.01	0.44	[−13.24, 29.27]	–0.05	0.96	[−2.56, 2.45]	–3.73	0.40	[−12.83, 5.37]
T_2_ (ms)	–3.21	0.042	[−6.29, −0.13]	0.32	0.087	[−0.05, 0.69]	0.64	0.37	[−0.82, 2.10]
OEF-Li-Bush (%)	0.015	0.037	[0.0010, 0.029]	–0.0021	0.0076	[−0.0036, −0.0006]	–0.0024	0.47	[−0.0090, 0.0043]
OEF-Bush (%)	0.018	0.0027	[0.0071, 0.029]	–0.0023	0.00069	[−0.0034, −0.0011]	–0.0036	0.21	[−0.0094, 0.0022]
OEF-Li (%)	0.019	0.015	[0.0043, 0.034]	–0.0027	0.0018	[−0.0042, −0.0011]	–0.0032	0.37	[−0.011, 0.0042]
OEF-Bovine (%)	–0.033	< 0.0001	[−0.044, −0.021]	0.0028	0.0059	[0.00092, 0.0047]	0.0095	0.014	[0.0022, 0.017]
CMRO_2_-Li-Bush (μmol/100 g/min)	11.63	0.0055	[3.91, 19.35]	–1.32	0.0069	[−2.22, −0.41]	–2.25	0.25	[−6.28, 1.78]
CMRO_2_-Bush (μmol/100 g/min)	13.32	0.00042	[6.89, 19.76]	–1.39	0.0023	[−2.21, −0.57]	–3.04	0.10	[−6.76, 0.67]
CMRO_2_-Li (μmol/100 g/min)	13.67	0.0027	[5.45, 21.90]	–1.60	0.0023	[−2.54, −0.66]	–2.67	0.22	[−7.10, 1.76]
CMRO_2_-Bovine (μmol/100 g/min)	–14.93	0.0027	[−23.90, −5.96]	1.54	0.0098	[0.42, 2.65]	4.15	0.074	[−0.44, 8.74]

*Adjusted for age and sex.

## Discussion

In this study, we investigated brain physiological parameters in pediatric sickle cell patients. We found that CBF is higher in SCD patients and this compensatory response appears to be sufficient in offsetting a reduced hemoglobin concentration, because total oxygen delivery, DO_2_, was not different from controls. In terms of OEF and CMRO_2_, the present study conducted a comprehensive investigation of four different calibration models and found that the results are dependent on model used. When using sickle-specific calibration (Li-Bush model, Bush model, and Li model), there was a decreased OEF and CMRO_2_ in SCD participants compared with controls, while the bovine model showed the opposite results. Importantly, we found that the relationship between sickle cell trait controls and non-trait controls is not dependent on model used, and CMRO_2_ was significantly lower in the trait participants regardless the model used.

T_2_-based MR oximetry such as TRUST MRI has been exploited widely due to its non-invasive property, less model assumption, short scan time and high reproducibility (Lu and Ge, 2008; Lu et al., 2012; Xu et al., 2012; Jiang et al., 2018, 2021). However, inconsistent results have been reported on whether OEF and CMRO_2_ are increased, decreased or unaffected in sickle cell disease (Jordan et al., 2016; Bush et al., 2018b,^2021^; Juttukonda et al., 2019, 2020; Li et al., 2020; Václavů et al., 2020; Prussien et al., 2021; Vu et al., 2021). Although one cannot rule out the possibility that different SCD population across different studies or other technical aspects such as different CBF measurement techniques may affect the results, the differences can be partly attributed to the T_2_-oxygenation calibration model used in the OEF quantification: Bush et al. (2018b), Juttukonda et al. (2020), Václavů et al. (2020), and Vu et al. (2021) used the Bush model, and consistently showed a decreased OEF and CMRO_2_ in patients with SCD; Li et al. (2020) reported the Li model and showed no change in both OEF and CMRO_2_; Bush et al. (2021) reported the joint Li-Bush model but did not report comparison with healthy controls; Jordan et al. (2016) and Juttukonda et al. (2019) used bovine model and both reported increased OEF and CMRO_2_ in more diseased people; Prussien et al. (2021) used human HbAA model presented in Bush et al. (2017) and showed that increased OEF was associated with deficits in executive function in SCD.

The original bovine model was derived from bovine blood, which is thought to have similar magnetic properties as normal human blood (Lu et al., 2012). However, it was also speculated that the bovine model was calibrated in a relatively small hematocrit range (35–55%), which is much higher than the hematocrit level in SCD (Lu et al., 2012; Bush et al., 2018b). In addition, the sickled red blood cell has different cell shape, size, permeability and tend to aggregate, which will affect the T_2_-relaxation in a different way than normal blood (Clark and Rossi, 1990; Gibson and Ellory, 2002). The higher concentration of reticulocytes and HbF could also alter the magnetic property of the sickled blood (Feng et al., 2015; Liu et al., 2016).

Three sickle-specific models have been presented recently, the Bush HbS model, the Li HbS model and the joint Li-Bush model (Bush et al., 2018b,^2021^; Li et al., 2020). The advantage of these models is that they were directly calibrated using human sickled blood. However, these models also have the potential bias caused by the polymerization and cell lysing in the *ex vivo* studies, which may amplify the effect of sickled red blood cell on the T_2_-Y_*v*_ relationship. It should be mentioned that the Bush study was also conducted with a relatively narrow hematocrit range (28 ± 4%), and the resulting model did not have hematocrit dependence. Additionally, it should be pointed out that there are two important differences between the T_2_ reported in Li et al. (2020) and those reported using the TRUST sequence, i.e., those reported in Jordan et al. (2016) and Bush et al. (2018b). One is that T_2_ reported in Li et al. (2020) used a turbo field echo (TFE) acquisition, and this TFE-T_2_ is known to be systematically different from the standard T_2_ in that peripheral k lines in TFE T_2_ acquisition are under-estimated. In fact, a previous study has developed a correction method for TFE-T_2_ and showed that TFE-T_2_ contains under-estimation for T_2_ values that are typical in SCD patients (Jiang et al., 2019). A second point is that, in Li et al. (2020) the *in vivo* T_2_ and the in vitro calibration T_2_ were not entirely equivalent. Their *in vivo* T_2_ was acquired on flowing blood, thus the TFE effect on the blood spin magnetization is different from the effect on the stationary blood. The stationary blood, which has no inflow spin refreshing, suffers from a greater TFE saturation effect in peripheral k lines. They reported a relative low T_2_ value for SCD participants (60 ± 10 ms), lower than the 91.2 ms in the current study, 96.9 ms in Bush study, 77.5 ms in Jordan et al. (2016) and 88 ms in Václavů et al. (2020). Combining data from these two studies with different T_2_ measurements added more complexity to the problem. Also, the Bush model and Li-Bush model omitted the linear term compared with the original bovine model.

In this study, we tried to compare all four models within the same cohort. We also used individual HbS level to interpolate the sickled calibration plot and normal calibration plot to obtain the Y_*v*_ at an individual level, in order to consider the inter-subject difference in terms of their blood composition. Our results showed decreased OEF and CMRO_2_ in SCD patients using Li-Bush model, Bush model, and the Li-model, but increased OEF and CMRO_2_ when using the bovine model. In general, our results are consistent with previous studies when using the same calibration models. The differences between the bovine model and the other three models are major and could result in completely opposite conclusions. The differences among the remaining three models are relatively small. These findings suggest that the disagreement in previous findings was partly attributed to the calibration model.

Li et al. (2020) reported a much closer OEF values (38%) to the PET study (42–44%) (Herold et al., 1986) compared with the Bush study (24%). However, this could be largely attributed to the T_2_ measurement difference rather than the model difference (that is, T_2_ experimental measurement resulted in an over-estimation of OEF and the calibration model resulted in an under-estimation of OEF; and the combination of these two yielded OEF that are close to the expected values). In this study, we applied three HbS models on the same T_2_ values, and the estimated OEF were in the same range, suggesting that the Bush model and Li model do not differ too much.

Without a gold standard measure (e.g., direct blood sampling from jugular veins), it is not possible to definitely determine which model results reflect the actual true physiology. However, using the results of comparison between trait and non-trait controls, one may gain some insight on the plausibility of the findings. We emphasize that trait controls were found to have a lower CMRO_2_ compared to non-trait controls for all four models. Since trait participants are expected to manifest a milder degree of sickle cell pathophysiology, it is reasonable to speculate that SCD patients should also have a lower CMRO_2_. These notions support the use of a sickle-specific calibration models in SCD T_2_-Y_*v*_ conversion, however, all three of the existing models have their own limitations as stated above. Furthermore, in vitro calibration experiment in sickle cell blood may suffer from cell lysis during oxygenation maneuver or other biochemical changes (e.g., accumulation of methemoglobin). Thus, we propose that an ideal calibration method for SCD OEF estimation would be to conduct calibration *in vivo* on a subject-specific fashion. This could be conducted by performing blood T_2_ measurement in a superficial vein in the arm, followed by venous blood sampling to measure the oxygenation level. A two-point calibration could be achieved by adding arm compression to restrict blood flow (which will alter blood oxygenation) or applying hyperoxia inhalation. These studies should be considered in future investigations. We would like to emphasize that due to small group size, the statistical results for the comparisons between trait and non-trait groups in this study should be interpreted with caution. This observation should be viewed as a preliminary finding only, and its value is to point out a potential direction for future research to verify in a larger cohort.

The lower OEF and CMRO_2_ found in patients with lower hemoglobin using the sickle-specific models is consistent with a previous study using nitrous oxide method, which showed reduced cerebral arterio-venous oxygen difference and CMRO_2_ in SCD patients (Heyman et al., 1952). Another PET study reported no significant difference in OEF and CMRO_2_ between groups, but the authors attributed it to a small sample size (Herold et al., 1986). One possibility of the reduced OEF and CMRO_2_ is that the high flow velocity drives down the oxygen extraction ability, which leads to the diminished oxygen metabolism. The mismatch between increased CBF and decreased OEF could be due to decreased capillary transit time (Juttukonda et al., 2020) or excessive CBF (Bush et al., 2018b) in arteriovenous shunting. On the other hand, overt stroke and SCI are often reported in SCD patients (Miller et al., 2001; Prengler et al., 2002). The decreased oxygen metabolism could also be a protective mechanism, which decreases the energy need in order to avoid significant loss of energy supply in the situation of ischemia. In this study, we did not find a significant cognitive impairment in SCD participants, despite the observation of a potential CMRO_2_ difference. It may be that brain functional measures such as CMRO2 are more sensitive than neuropsychological tests in terms of detecting early brain abnormalities. Follow-up studies would provide more insight into the neurodevelopmental consequences of the oxygen metabolism impairment.

To our knowledge, there are few studies examining the oxygen metabolism in pediatric SCD patients. Existing studies on pediatric patients all used susceptometry-based method and reported incongruent results, i.e., increased or decreased OEF and CMRO_2_ (Fields et al., 2015, 2018; Croal et al., 2019), which may be related to the model assumption in the technique. In terms of magnetic susceptibility properties of SCD blood, one recent study systematically investigated the susceptibility difference between deoxy- and oxyhemoglobin, suggesting that HbS and HbA blood may have little differences in their susceptibilities (Eldeniz et al., 2021). Our study is the first to apply the T_2_-based oximetry on pediatric patients and confirmed a similar change of OEF and CMRO_2_ in SCD children compared with adults.

There are several limitations in our study. First, the sample size is relatively small and the study population is heterogeneous. Although the heterogeneity may make the comparison between SCD and control more complex, it could add more dynamic range of the parameters and provide more insight into the disease, rather than just focus on the most severe type. Considering the small sample size, it is hard to investigate how cerebral physiology is different for different phenotypes. More comprehensive studies with larger sample size would help to further understand the oxygen homeostasis in pediatric sickle cell disease. Second, the sickle-specific models are calibrated in adult HbS blood, which may be different than that of the pediatric population, considering different concentrations of HbS, HbF and reticulocytes. For example, Liu et al. (2016) reported that neonatal blood has a longer T_1_ and T_2_ compared with adult blood at the same hematocrit and oxygenation level, potentially due to different molecular structure and oxygen biding affinity of HbF. On the other hand, higher concentration of reticulocytes which has high molecular weight micro-organelles such as ribosomes could shorten the blood T_2_ (Feng et al., 2015). Thus the adult HbS model may not be suitable for pediatric population. Further studies may be needed to better establish the T_2_ calibration model in pediatric patients. Third, the TRUST MRI is a global measurement, which cannot provide regional information of the oxygen extraction. Future application of regional specific method may be useful.

## Conclusion

We examined CBF, oxygen delivery, cerebral oxygen extraction, and metabolism in pediatric SCD patients. SCD patients revealed an elevated CBF, but normal oxygen delivery. The results of OEF and CMRO_2_ were dependent on the calibration models used, and different models yielded opposite results. On the other hand, trait controls were found to have lower CMRO_2_ than non-trait controls, regardless of the model used, which may help shed some light on the model selection in SCD oxygenation studies.

## Data Availability Statement

The raw data supporting the conclusions of this article will be made available by the authors, without undue reservation.

## Ethics Statement

The studies involving human participants were reviewed and approved by the Institutional Review Board (IRB) of Johns Hopkins University. Written informed consent to participate in this study was provided by the participants’ legal guardian/next of kin.

## Author Contributions

ZL, JC, HL, and EL designed the research study and wrote the manuscript. ZL, PL, HL, and EL performed the MRI experiments and collected the data. TM, EL, and AC designed and/or performed the cognitive examination. ZL, TM, DJ, KS, and AT analyzed the data. JC assisted with the interpretation of data. All authors edited and approved the manuscript.

## Conflict of Interest

EL served on an advisory board for Novartis for sickle cell disease therapeutics. JC is an inventor and a named party on a patent and licensing agreement to ImmunArray through Johns Hopkins for a panel of brain biomarkers for the detection of brain injury, and also holds a patent for aptamers as a potential treatment for sickle cell disease. The remaining authors declare that the research was conducted in the absence of any commercial or financial relationships that could be construed as a potential conflict of interest.

## Publisher’s Note

All claims expressed in this article are solely those of the authors and do not necessarily represent those of their affiliated organizations, or those of the publisher, the editors and the reviewers. Any product that may be evaluated in this article, or claim that may be made by its manufacturer, is not guaranteed or endorsed by the publisher.

## Footnotes

1
www.MRICloud.org
